# The Effect of Extracellular Vesicles on Thrombosis

**DOI:** 10.1007/s12265-022-10342-w

**Published:** 2022-11-28

**Authors:** Youfu He, Qiang Wu

**Affiliations:** 1 Medical College, Guizhou University, Guiyang, Guizhou Province, China; 2 Department of Cardiology, Guizhou Provincial People’s Hospital, Guiyang, Guizhou Province, China; 3 Guizhou Provincial Cardiovascular Disease Clinical Medicine Research Center, Guiyang, Guizhou Province, China

**Keywords:** Acute thrombosis, Extracellular vesicles, Tumor-secreted tissue factor, Inflammation, Endothelial cells

## Abstract

The risk of cardiovascular events caused by acute thrombosis is high, including acute myocardial infarction, acute stroke, acute pulmonary embolism, and deep vein thrombosis. In this review, we summarize the roles of extracellular vesicles of different cellular origins in various cardiovascular events associated with acute thrombosis, as described in the current literature, to facilitate the future development of a precise therapy for thrombosis caused by such vesicles. We hope that our review will indicate a new horizon in the field of cardiovascular research with regard to the treatment of acute thrombosis, especially targeting thrombosis caused by extracellular vesicles secreted by individual cells. As more emerging technologies are being developed, new diagnostic and therapeutic strategies related to EVs are expected to be identified for related diseases in the future.

## Introduction

There is a high risk of developing cardiovascular events from acute thrombosis (AT), including acute myocardial infarction (AMI), acute stroke, acute pulmonary embolism, and deep vein thrombosis. The mortality rate of AT is very high, with no effective preventive measures existing for AT-related pathologies in arteries and veins, primarily because the underlying mechanism of thrombosis remains unclear.

Thrombosis is a complex pathophysiological process. In addition to the physical factors of high shear stress associated with arterial thrombosis and low shear stress associated with venous thrombosis, a number of specific pathophysiological processes that can also affect thrombosis have been recognized in recent years. Such processes include alterations in platelet function [1], neutrophil extracellular network formation [2] (neutrophil extracellular traps, NETs), activation of coagulation factors [1], altered vascular structure [3], increased oxidative levels [4], inflammatory response [5], and lipid metabolism [6]. However, contemporary medical research often overemphasizes the therapeutic role of anticoagulation and antiplatelets in thrombotic events, while failing to explore other cellular and molecular-related mechanisms of thrombosis. In particular, the role of vesicle-like substances secreted by various types of cells in the development and treatment of AT is a blind spot in current cardiovascular research. These substances include microvesicles (MVs, ~ 0.05–1 μm in diameter [7]), exosomes (~ 30–150 nm in diameter [8]), extracellular particles (microparticles, MPs, about 100–1000 nm in diameter [9]), and apoptotic bodies (apoptotic bodies, about 1–4 μm in diameter [9]).

The limited number of clinical studies suggests that extracellular vesicle (EV)-based treatment combines the advantages of hypoallergenicity, high efficiency, and precision treatment [10, 11]. Moreover, recent research has also focused on the relationship between exosomes and thrombosis. For example, a study by Fabrice et al. [12]confirmed that glypican-1-positive EVs can be used as a marker for early thrombosis in pancreatic cancer. Here, we review the role of EVs of different cellular origins in various cardiovascular events associated with AT.

## Secretion and Activation of Extracellular Vesicles from Various Cell Sources

There is no definitive conclusion that different cells secrete EVs with different markers. However, few studies have found that some cells secreting EVs may carry the surface markers of those cells [13]. New studies also confirm that, for example, CD41a-positive EVs in plasma are most likely secreted by platelets [14], providing some theoretical basis for our discussion (see below). However, the pathways by which EVs are activated and released are not the same. In the following, we address the involvement of different cell-activated and -released EVs in the regulation of the coagulation function.

### *Platelets*

#### The Thrombin and Phosphatidylserine-Related Pathway

It is generally believed that thrombus formation is mainly associated with platelet activation and platelet-derived extracellular vesicles (PEVs). Through various pathophysiological effects (e.g., inflammatory stimulation, tumor, oxidative stress, immune response, and high glucose stimulation [15–18]), thrombin acts on the platelet plasma membrane and cytoplasm to promote the formation of PEVs [19]. Moreover, hypoxic stress stimulates platelets to synthesize fibrinogen activator inhibitor-1 (plasminogen activator inhibitor type-1, PAI-1) and promotes the production of EVs [20]. These EVs provide anionic phospholipids, such as phosphatidylserine (PS), which can support the coagulation cascade. In addition, EVs released by platelets can participate in the inflammatory and coagulation processes brought on by phosphatidylserine exposure [21]. For example, a significant increase in EVs was observed in patients with COVID-19 infection, whereas the levels of PS-exposed PEVs were significantly higher only in patients with the non-severe disease [22]. Notably, D-dimer is a marker of thrombosis, although its levels do not clearly correlate with platelet activation [22]. Following EV formation, they are released from platelets under the regulation of cytoplasmic Ca^2+^ signaling and calmodulin activity, causing PS exposure on platelets and EVs. This phenomenon also induces, in turn, the activation of platelets and their EVs, with corresponding procoagulant effects [23]. Moreover, cholesterol-rich lipid rafts provide a platform for platelet-associated receptors and calcium-associated signals to activate platelets and induce EV shedding [24]. Activators (e.g., arachidonic acid, adenosine diphosphate (ADP), collagen, thrombin, and calcium carrier A23187) can also promote the release of PEVs, with thrombin specifically inducing the formation of particles from the platelet plasma membrane and cytoplasm, and from intracellular structures [19]. PS-exposed EVs can have a positive feedback effect on thrombin. In addition to their direct involvement in thrombin translation [25], in vivo trauma experiments confirmed that trauma led to a significant release of platelet EVs into circulation, where they induce significant thrombin production, increase platelet aggregation, reduce the number of bleeds, and decrease uncontrolled bleeding. Therefore, it has been suggested that EVs contribute to enhanced thrombosis and are recruited to the site of thrombosis [26].

#### Tissue Factor-Related Pathways

Tumor-secreted tissue factor (TF or CD142) can also activate coagulation and trigger venous thrombosis (via its action on circulating EVs) [27]. Running has been suggested to significantly reduce the TF content of platelets, indicating that cardiovascular risk may be reduced (at least temporarily) by reducing TF-stimulated thrombosis [28]. A recent study showed that patients with AMI have significantly increased PCM levels in their circulating blood compared to the general population, which may be related to TF expression as indicated by EVs [29]. TF is a major trigger of fibrin formation and a cellular initiator of the exogenous coagulation cascade reaction, playing an important role in intravascular thrombosis. The EVs carrying TF are among the most procoagulant [30]. The mechanism of platelet activation triggered by intestinal cancer cells is dependent on the expression of cancer cell TF, with thrombin production activating protease-activated receptor 4 (PAR4) on platelets, thereby promoting ADP and thromboxane A2 (TXA2) release [31]. This is subsequently accompanied by remodeling of the phospholipid and charge membrane structure on the surface of platelet-derived microparticles (PMPs), enabling them to exhibit significant procoagulant activity, further increased by the presence of TF [32]. These results suggest that TF-related activation of PEVs is a main pathway of EV activation. However, according to clinical studies, EVs account for approximately 84% of PS-exposed EVs, whereas EVs carrying TF account for approximately 2.5%. Hence, phospholipids (e.g., PS) are essential for TF procoagulant activity, with considerable synergy between the two compounds [33].

#### The Glycoprotein Ibα–von Willebrand Factor-Related Pathway

**Fig. 1:**
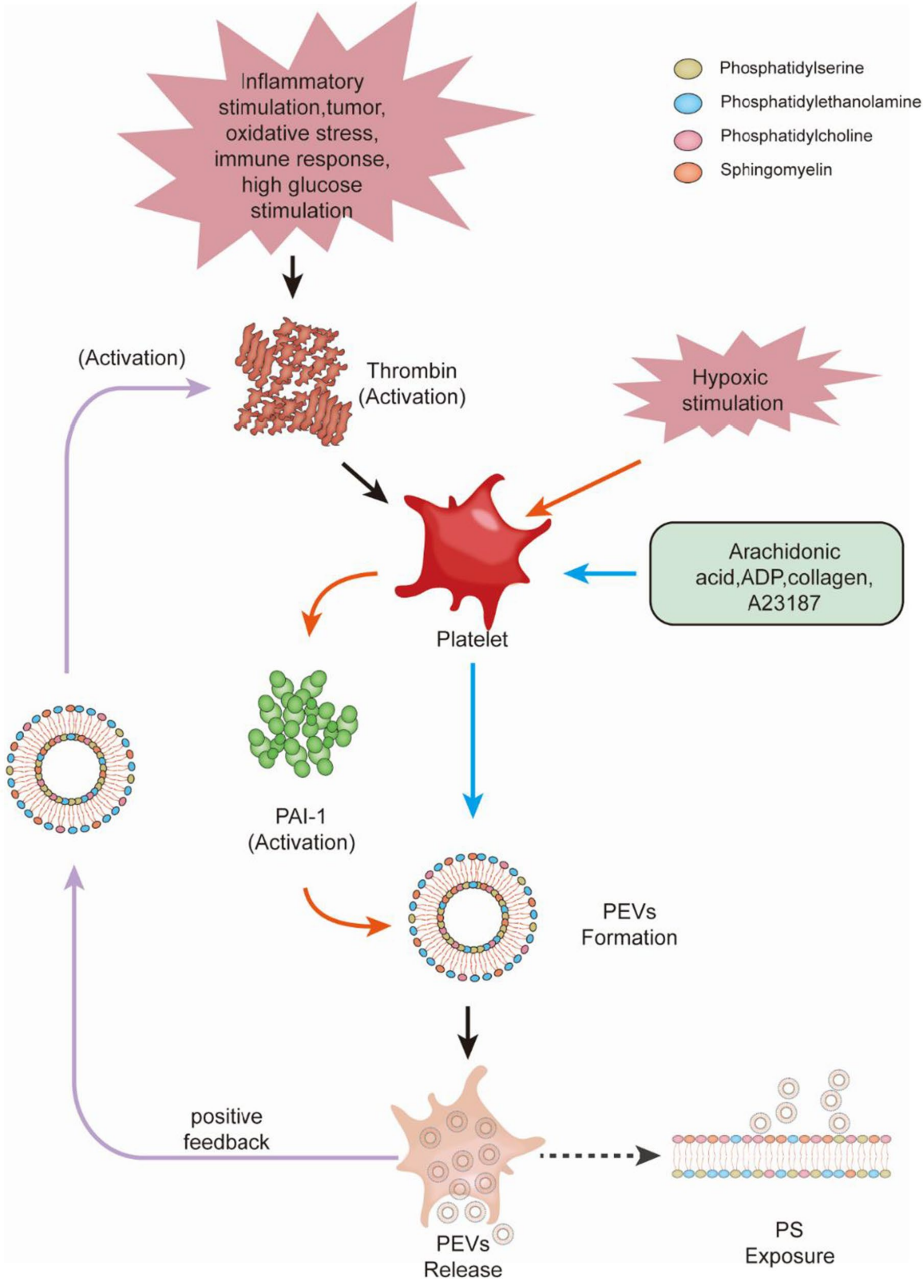
Platelet activation and the release of extracellular vesicles

When platelets are activated by agonists (e.g., inflammatory factors in whole blood), they shed EVs derived from the corresponding stimuli, which rapidly and preferentially bind to blood monocytes. The binding of PEVs to monocytes is caused by P-selectin-dependent adhesion [34] and stabilized by the binding of PS [35]. However, these interactions can lead to the progressive transfer of platelet adhesion glycoprotein receptors (glycoprotein Ibα, GPIbα) on platelets to monocytes. The GPIbα + monocytes can then be bound by von Willebrand factor (vWF) and transforming growth factor-β1 (TGF-β1)-treated endothelial cells (ECs), to be recruited and activated to induce vWF expression [36]. In both models, monocyte adhesion is cleared by GPIbα function-blocking antibodies. In in vivo experiments, GPIbα + monocytes adhered to TGF-β1-stimulated vascular smooth muscle, whereas in the apolipoprotein E (APOE) − /– atherosclerosis model, GPIbα + monocytes adhered to carotid arteries. In trauma patients, monocytes carry platelet markers within 1 h of injury, at levels that correlate with the severity of the trauma, leading to the clearance of monocytes from circulation [35]. This EV-associated thrombotic inflammatory pathway is distinct from the first two EV activation pathways in that EVs transfer platelet adhesion receptors to monocytes for their reaggregation in large and small vessels. Related studies have found that inhibition of platelet adhesion to the arterial wall may induce thrombocytopenia and significantly reduce monocyte trafficking, along with the prevalence of atherosclerotic disease in APOE-knockout mice, whereas inhibiting platelet adhesion to the arterial wall also significantly inhibits local platelet production [37]. Figure 1 shows a schematic diagram illustrating the process of vesicle activation and release within platelets.

### Leukocytes

Little is known regarding the role of various types of leukocyte-derived thrombosis. Previous studies have found that high glucose stimulation [15], local injury [38], inflammatory stimulation [39], and oxidative stress [40] are responsible for promoting the formation of leukocyte-derived EVs. Moreover, monocyte-derived microvesicles (MDMVs) express large amounts of TFs and are involved in thrombosis and cardiovascular disease. Conversely, HIV stimulation of monocytes inhibits tetherin expression and promotes the production of MDMVs, thereby increasing the risk of cardiovascular disease in patients with HIV. This occurs because overexpression of tetherin in monocytes leads to morphological changes in the lower pseudopods of EVs, which in turn affects their activity. In turn, the knockdown of the tetherin gene resulted in a significant increase in the number of circulating EVs and a significant decrease in bleeding arrest time in mice, which is a strong indication of the important role of EVs in the coagulation system [41]. Similarly, it has been found that the prothrombotic state in patients with cardiovascular disease is associated with increased TF expression in monocytes and elevated levels of circulating EVs, and that interleukin-33 (IL-33) induces differential TF expression and activity in monocyte subpopulations, further promoting the release of EVs [42]. EVs produced after stimulation by local injury are enriched in NADPH oxidase 2 (NOX2) complexes. These NOX2 complexes are retrogradely transported to the cell body and thus oxidize intracellular PTEN through a β1 kinesin-dependent mechanism, leading to its inactivation. The complexes subsequently stimulate PI3K phosphorylation-Akt signaling to produce the corresponding effect [39]. Concurrently, EVs in patients with cardiovascular disease significantly inhibit in vivo activation of endothelial nitric oxide synthase (eNOS), the bioavailability of nitric oxide (NO), and tissue plasminogen activator (t-PA) production, which promotes atherosclerosis and thrombosis [43].

In patients with diabetes, a high-sugar diet increased plasma levels of EVs, endothelium-derived EVs, and monocyte-derived EVs, with the procoagulant effects of these EVs associated with their negative surface charge and PS, but not with TF [15]. Similarly, the NET-associated MPs in the blood of patients with cerebral infarction are dominated by PS-modified procoagulant pathways. These MPs promote NETs to provide a platform for PMPs and increase thrombin and fibrin formation [40]. In addition, cellular adenosine triphosphate (ATP) signaling plays an important role in the secretion of MPs by cells such as macrophages, and extracellular ATP signaling can induce the activation of TF procoagulant activity via recombinant purinergic receptor P2X and ligand-gated ion channel 7 (P2X7), which induces TF procoagulant activity [44]. When macrophages are stimulated by ATP signaling, these P2X7 receptors subsequently cause uncoupling of the thioredoxin/TRX reductase (TRX/TRXR) system and activate the inflammasome via endosomally generated reactive oxygen species (ROS). TRXR and inflammatory vesicle activity promote filopodia formation, the cellular release of reduced TRX, and the production of extracellular sulfhydryl pathway-dependent EVs [45]. Subsequently, TF^+^ MPs are released from cells in macrophages and smooth muscle cells under the control of ROS signaling [44].

### Macrophages

In addition to leukocytes, macrophages play an extremely important role in the process of thrombosis. In a study on inflammatory vesicles caused by Gram-negative bacteria and their procoagulant effects, researchers found that with the activation of inflammatory vesicles, macrophages release TF-containing EVs, which in turn mediate thrombogenesis [46]. Additionally, inflammasome-induced activation of an intracellular caspase-1/calpain cysteine protease cascade degraded filamin, thereby severing bonds between the cytoskeleton and TF, the cell surface receptor responsible for coagulation activation. This cascade enabled TF trafficking from rafts to filopodia and ultimately onto phosphatidylserine-positive, highly procoagulant MPs, or EVs [45]. Furthermore, in an animal experiment on mice, it was shown that activation of these macrophage-derived microparticles (MΦMPs) may be associated with P2X7 receptor signaling [44]. Moreover, researchers have suggested that thrombosis in patients with cardiovascular disease may also be associated with MΦMPs and TF, and that OxLDL induces the production of TF-enriched MΦMPs, a process dependent on caspase3/7 and CD36, which can be inhibited by statins (mevastatin) [38].

### Erythrocytes

Red cell extracellular vesicles (REVs), also known as red cell microparticles (RMPs), are produced by erythrocytes, although the reason for their production remains unclear. In physiological conditions, red blood cell (RBC)-derived EVs compose 4–8% of all circulating EVs [47]. Erythrocytes eliminate hemichromes by vesiculation in response to oxidative stress, protecting other healthy erythrocytes at the expense of the cells themselves [45]. In 1976, REVs were initially thought to be dust-like substances produced by platelets; hence, their function was unresolved [48]. Later, based on various thrombotic events following allogeneic transfusion [49], they were found to be effective in stopping hemorrhaging without producing thrombi in vivo and to possibly constitute safe and effective new hemostatic agents [50]. These particles, similar to PEVs, exhibit mainly phospholipid-dependent procoagulant activity, with their procoagulant effect proportional to the particle dose [51]. The procoagulant effect of REVs is extremely strong. Moreover, there is a significant increase in TF expression in monocytes and plasma after incubation with REVs, with a subsequent time- and concentration-dependent increase in TF expression and consequent induction of IL-1β, IL-6, and IL-8 expression, which in turn activates the corresponding inflammatory pathways [52].

Early studies on REVs in the cardiovascular field were mainly limited to respiratory diseases, in which it was shown that patients with pulmonary hypertension are prone to pulmonary embolism mainly owing to significantly increased PS levels in REVs and PEVs compared to those in normal subjects and that PS exposure further induces subsequent coagulation, which in turn leads to pulmonary artery embolism formation [53]. REVs in patients with obstructive sleep apnea are thought to significantly impair endothelium-dependent diastolic function, where they may play a role in EC functional impairment and subsequent thrombosis by blocking eNOS phosphorylation. The latter happens through inhibition of the PI3K/Akt pathway and enhancement of EC endothelin-1 (ET-1) expression upon activation of the Erk1/2 pathway, which plays an extremely important role in EC functional impairment and subsequent thrombosis [54]. In recent years, other branches of cardiovascular disease research have also started to investigate REVs. In sports medicine, a recent study found that following heavy exercise (10-km-long-distance running), the deformability of REVs was enhanced and blood viscosity was further reduced. In the same study, no significant effect on erythrocyte senescence and the lipid membranes of REVs in circulating blood was observed [55]. After 6 weeks of endurance training, total circulating blood erythrocytes, PS exposure, and the amount of REVs decreased, suggesting that exercise may reduce the risk associated with thrombosis [56]. In a coronary study, PS enriched in REVs exhibited anticoagulant properties only in healthy individuals but not in non-healthy populations. The study also noted a significant increase in the total number of REVs in patients with the acute coronary syndrome (ACS). Thus, this may serve as a potential marker of persistent thrombosis in patients with ACS [57]. Sudnitsyna et al. [47] also found that oxidative stress can significantly increase the level of REVs in circulating blood. Furthermore, oxidative stress is an important factor affecting thrombosis. Together, these findings strongly support the involvement of REVs in thrombosis.

### Endothelial Cells

In the cardiovascular field, ECs have received insufficient attention with regard to thrombus formation. The few findings on EV secretion by ECs have shown that Rho-kinase may be involved in endothelial EV formation by regulating cytoskeletal structure [58]. In actual clinical studies, analysis of circulating blood EVs in stroke populations has shown that patients with stroke have significantly increased levels of circulating blood EVs, and that these increases are associated with the activation of EVs, ECs, and platelet cells [59]. Moreover, smoking significantly increases the levels of PMPs and MVs secreted by ECs, suggesting that endothelial damage and platelet activation are potentially related to thrombosis [60].

The activation and release of EVs secreted by ECs, and the TFs therein, constitute major contributors to the initiation of local and systemic coagulation. The transport and release of TFs on EVs coincide with the release of cell adhesion receptors, including integrin beta1 heterodimers, which control the transport of the TF-activating factor VIIa (FVIIa) complex. Activation of the TF signaling chaperone PAR2 also induces the release of integrin beta1 and TF-enriched EVs from ECs [61]. On ECs, FVIIa specifically induces the formation of TF complexes with integrin α5β1 [61]. These TF-FVIIa complexes are major activators of coagulation, and elevated levels of TF-dependent procoagulant activity hosted by EVs can be detected in patients at increased risk of thrombosis [62]. FVIIa interacts with integrin β1 to control intracellular transport associated with EVs and regulate TF release from procoagulant EVs [61].

Alternatively, thrombin/CD40L and lipopolysaccharide (LPS) also stimulate EC activation and the secretion of EVs containing matrix metalloproteinase-10 (MMP-10) and CD40L, which in turn produces a procoagulant effect. Thrombin/CD40L and MMP10-containing MVs have a synergistic effect that is dependent on the p38 mitogen-activated protein kinase (p38 MAPK) and c-Jun N-terminal kinase-1 pathways [63]. After thrombin stimulation of ECs, the tumor necrosis factor (TNF)-related apoptosis-inducing ligand (TRAIL)/TRAIL receptor 2 (TRAIL-R2) complex is activated by initiating the recruitment of bridging proteins, and the activation of nuclear factor kappaB (NF-κB), to mediate the release of MPs from ECs. Moreover, TRAIL regulates the expression of thrombin-induced intercellular adhesion molecule-1 (ICAM-1) and IL-8 through activation of the downstream pathway of NF-κB [64].

### Tumor Cells

As early as 2012, it was found that TFs overexpressed on circulating EVs could activate coagulation and trigger venous thrombosis in a xenograft mouse model [27]. It has also been shown that EVs secreted by tumor cells with negatively charged phospholipids have maximal procoagulant activity [65]. In a subsequent study, EVs secreted by tumor cells could lead to a procoagulant shift of ECs, which subsequently express TFs and promote thrombin production. This phenomenon of altered procoagulability of endothelial cells consequent to EVs secreted by these tumor cells can be inherited even in endothelial cell offspring that are not exposed to EV stimulation [66]. Tumor cells can also exacerbate the local thrombogenic effect by releasing granulocyte-colony stimulating factor (G-CSF) into the bloodstream, which stimulates circulating neutrophils to form NETs [67].

More in-depth studies have shown that tumor cell (pancreatic cancer cell)-derived MVs are enriched with TFs, tissue factor pathway inhibitors (TFPI), and integrins αvβ1 and αvβ3, which induce thrombogenic effects by activating platelets and fibrinogen [68]. Moreover, these TF-enriched EVs activate human platelets both in vitro and in vivo and induce platelet aggregation in a TF- and thrombin-dependent manner via PAR4 [69].

#### Other Cells

In addition to the aforementioned cells that are well known, adipocytes can secrete EVs containing PS and TF without any conditional activation and participate in procoagulant responses related to exogenous pathways [70]. The vesicles secreted by MSCs, the most frequently used cells in EV-related therapy, contain PS and TF as well [71]. This finding may alert both medical professionals and researchers to the need for a high level of vigilance for thrombosis as a side effect. There is also evidence that EVs secreted by endothelial progenitor cells can promote thrombus lysis and recanalization via miR126 enriched within them in a model of lower extremity deep vein thrombotic disease [72].

The conditions of activation and the pathways involved are relatively clear for some of the mentioned cells, while others have been very poorly studied. We recommend to address the key mechanism of “cellular activation and EV release” in subsequent studies. Disentangling this mechanism requires to distinguish between different EVs secreted by different cells, and this will serve as a bridge between “EVs release” and “thrombosis” to find a new therapeutic target.

## Effector Substances of Various Cell-derived Extracellular Vesicles and Their Mechanisms

As mentioned earlier, various cells are activated to release different EVs to participate in thrombosis. These EVs are known to contain different effectors. In the following, we further discuss these effectors.

### Protein

**Fig. 2:**
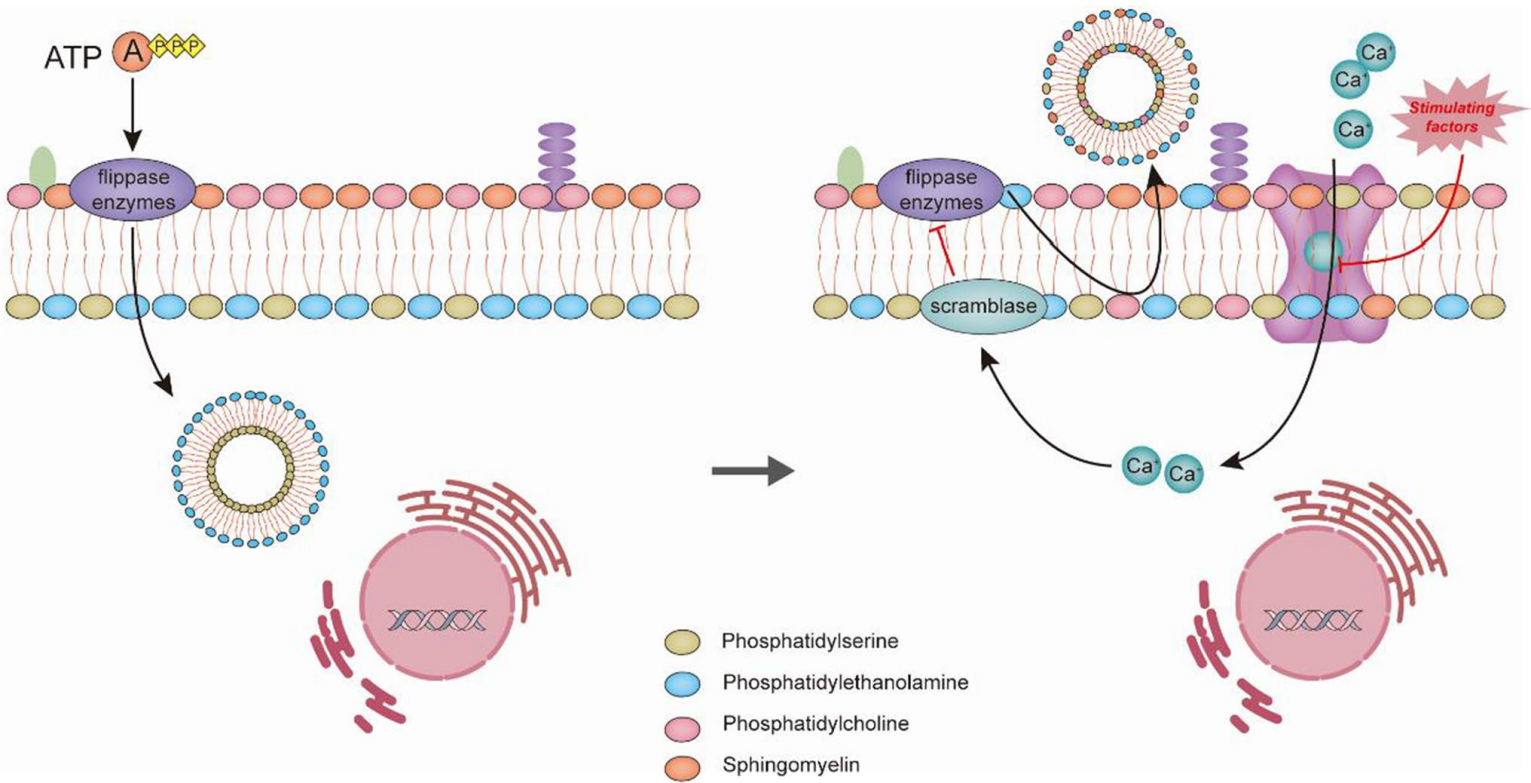
Extracellular vesicle entry and exit processes

EV bodies are mainly heterogeneous membrane-bound phospholipid vesicles actively secreted by living cells [73]. The phospholipid bilayer of the cell membrane is asymmetrically distributed, with the outer cell membrane being rich in phosphatidylcholine and sphingolipids, whereas the inner layer is mainly formed by PS and phosphatidylethanolamine [74]. This lipid distribution is mainly maintained by the action of flippase enzymes. Under specific stimuli, the inward flow of cytosolic Ca^2+^, the activation of nonspecific lipid transport proteins (scramblase), and the inhibition of the expression of flip-flop enzymes [75], which disrupts this asymmetry, lead to the redistribution of phospholipids across the membrane bilayer and promotes the outward blistering of the fine membrane to form tiny vesicle bodies and release them outward (outgrowth). This is the main process for the release of EV bodies [76]. A schematic of the process of vesicle entry and exit from the cell is shown in Fig. 2. Because these secreted EVs do not possess the organelles of living cells, they cannot actively produce genes and proteins, acting more as carriers of cellular components. Depending on the source, vesicles from different cell types may contain different source-associated proteins (e.g., Rab GTPase, SNAREs, annexins, and flotillin), some of which will also be involved in vesicle body production and secretion (e.g., alix and TSG101) [77]. Membrane proteins known to be involved in the aggregation of cell membranes or endosomes into tiny structural domains are usually also enriched on these tiny vesicles, including tetraspanins. These proteins constitute a family of more than 30 proteins (including CD63, CD81, CD82, CD53, and CD37) and contain four transmembrane structural domains [78]. As these proteins are expressed on EVs (including exosomes, MPs, and MVs), they are generally detected as specific markers of EV [79].

In addition to the various proteins that constitute EV bodies, the main effector substances that are enriched on the vesicle bodies (secreted by various cell types) are coordinated with known mechanisms of thrombogenesis, and can be broadly classified as follows: altered blood composition, altered coagulation and fibrinolytic system activity, endothelial dysfunction, and inflammatory response [80]. However, a review of recent literature did not find any clear evidence that substances enriched in EVs in vivo affect blood composition or blood viscosity, including all types of blood cells, suggesting this as a potential new direction of research.

## Activation of the Coagulation System and Altered Fibrinolytic System Activity

Various types of EV bodies are enriched with TFs, which, in addition to promoting the formation and activation of EVs, are also the main effector substances. Moreover, circulating EVs are enriched in large amounts of procoagulant phospholipids (PPL), which are key components in promoting blood coagulation activity, and the pathological hypercoagulable state. These substances are the main cause of thrombosis in some patients [81]. PPL in circulating EVs stimulates thrombin activation with TF and forms FVIIa/antithrombin complexes, and may be involved in the regulation of activated protein C activity [81]. EVs can express various bioactive lipids, proteins, and nucleic acids that stimulate thrombus formation and promote vascular activation through a dual mechanism of TF-dependent and -independent mechanisms, thus playing a potential pathogenic role in various thrombus-prone diseases [82]. For example, in a model of cerebral ischemia, rat platelet-derived exosomes increase PAI-1 in ECs by promoting endothelial permeability and TF protein levels, which in turn promote prothrombotic effects and induce thrombosis [83]. PS + and TF + EV subpopulations were also detected in patients with atrial fibrillation and were found to determine their procoagulant effects [84]. Similarly, in patients with stem cell transplantation, MVs secreted by Mesenchymal stem cells (MSCs) promote a hypercoagulable state of blood mainly through TF and PS [71]. Furthermore, in a study of a model of thrombotic meningoencephalitis disease caused by *Histophilus somni*, researchers found that bovine brain ECs secreted large amounts of vWF-rich EVs, which contribute to fibrin clot formation and also increase TF activity in a positive feedback-regulated manner, exacerbating thrombosis [85]. It was also demonstrated that MVs released from platelets in the blood of patients with type 2 diabetes mellitus (T2DM) carry a large number of platelet-active substances such as P-selectin, GPIb/CD41, and GPIIb/IIa. These substances, while activating platelets in reverse, also promote platelet repertoire and PS exposure, which in turn induces thrombosis [86, 87] Moreover, Birke et al. [88] identified 585 EV-related proteins by analyzing EVs secreted by airway epithelial cells (BEAS-2B) under basal conditions and upon exposure to cigarette smoke extract (CSE), using a Nano-LC–MS/MS system. Functional enrichment analysis revealed that 24 proteins of the hemostatic pathway (Table 1), including TF, were significantly upregulated in CSE-EVs, all of which were associated with TF activity. Moreover, these proteins were dependent on TF and PS, which significantly promoted coagulation. These results suggest that the main effectors within EVs are associated with TF and PS.

**Table 1:** Main effector proteins in various types of EVs

Cell source	EV type	Protein
Airway epithelial cells (BEAS-2B) [88]	CSE-EVs	AES, MPDU1, IL16, DNAJC8, C19orf33, LRRC59, PPBP, GPS1, MED11, S100A9, MYL9, YWHAH, HPS1, RGL4, LCN2
Circulating blood in patients with the peripheral arterial disease [90]	AnnexinV^+^EVs	AES, MPDU1, IL16, DNAJC8, C19orf33, LRRC59, PPBP, GPS1, MED11, S100A9, MYL9, YWHAH, HPS1, RGL4, LCN2

However, the thrombin activation induced by EVs can be inhibited by the overexpression of AnnexinV, whereas TF antibodies have no relevant effect. This suggests that under certain specific pathological conditions, EVs may promote coagulation under PS exposure, whereas the effect of TF is minimal [89]. Among them, annexinV proteins, mainly expressed in PEVs, can be detected in large quantities in the circulation of patients with the peripheral arterial disease (PAD), suggesting that these proteins may be associated with the procoagulant activity of platelet-free plasma. Analysis of annexinV-positive EVs revealed that they are enriched in various types of proteins that participate in the coagulation pathway (Table 1), such as serum calprotectin S100A8/A9, the detection of which may increase the accuracy of amputation risk prediction in patients with PAD [90].

Similarly, Morad-Rémy et al. [91] compared the molecular composition, procoagulant, and immunogenic properties of exosomes, EVs, and apoptotic vesicles secreted by B16 melanoma, and found that different types of vesicles express different surface and cytoplasmic molecules. These molecules include tetrapeptides, integrins, heat shock proteins, and histones. Moreover, in-vitro coagulation assays showed that membrane-derived vesicles (e.g., MVs and apoptotic vesicles) are more procoagulant than exosomes carrying TF and PS [91].

Dzhigangir et al. [92] revealed another link in the role of EV bodies in activating the coagulation cascade reaction. Specifically, the lipid bilayer membrane of MVs has diffusion barriers to the three-dimensional aggregation of fibers and produces spatial restrictions on fiber elongation. Alternatively, the high concentration of plasma fibrinogen adsorbed on the surface of liposomes promotes the polymerization of fibrin. Moreover, such surface adsorption adsorbs to cause changes in fibrinogen secondary structure, which may underlie in part the abnormal thrombus morphology caused by EVs. Mancy et al. [93], studying the secretion of EVs by trophoblast cells, found that EVs are also enriched with a large number of antiphospholipid antibodies that target phospholipid-binding proteins such as beta-2-glycoprotein I (β2GPI), which in turn induces systemic thrombosis and increases the risk of obstetric complications such as recurrent miscarriage and pre-eclampsia. In addition, Nox-1 is enriched within PEVs, where they induce thrombosis while reverse-activating platelets by regulating ROS production downstream of glycoprotein VI (GPVI) [94].

### Endothelial Dysfunction

Consistent with the activation and procoagulant activity of ECs, the large number of TF proteins carried on circulating EVs, or EVs secreted from apoptotic ECs, induces the activation and secretion of EVs after activating PAR2 receptors on ECs [61]. These TF signals induce a coagulation cascade on ECs, allowing them to bind to factors IX and X, which promote thrombogenesis while leading to abnormal EC functionality [95]. In addition, following apoptosis of the ECs, the PS contained within the ECs is redistributed to the cell surface and the anticoagulant components on the cell surface (including thrombomodulin, acetyl heparan sulfate, and TF pathway inhibitors) are subsequently lost or depleted, producing procoagulant effects [96]. We, therefore, directed our attention to various types of EV bodies with regard to EC dysfunction.

The effect of vesicles of various cell sources on ECs is bi-directional. For example, tumor cell-derived exosomes may be enriched in angiogenic factors such as angiopoietin, IL-6, IL-8, vascular endothelial-derived growth factor (VEGF), tissue inhibitor of metalloproteinase 1/2 (TIMP-1/2), and other angiogenic factors, to activate EC function and enhance their activity to stimulate angiogenesis [97]. They may even be enriched with factors such as epidermal growth factor receptor (EGFR), VEGF receptor 2 (VEGFR-2), and ephrin type-A receptor 2 (EPHA2) to help ECs resist apoptosis [98]. Alternatively, in cardiovascular diseases, Osman et al. [99] found that circulating EVs induce EC disorders or even directly cause EC apoptosis by activating endoplasmic reticulum stress. Moreover, they found that circulating EVs not only contain eNOS [100] but also affect NO release from ECs [99], with impaired NO release highly suggestive of EC diastolic dysfunction [101]. Circulating EVs altered inflammatory factor release from ECs; however, there was no clear evidence that apoptosis and autophagy of ECs were affected [99]. Based on the available evidence, the effects of EVs on ECs appear to be driven either through NO-related pathways or prostacyclin synthesis pathways rather than through CD11a/CD18 adhesion molecules or the Fas/FasL pathway [102]. However, Zainab et al. [103] found that circulating MPs from patients with metabolic syndrome carry Fas ligands and low-density lipoprotein receptors, through which they induce mitochondrial function in ECs, causing a sequential increase in cytoplasmic and mitochondrial ROS. This in turn causes endoplasmic reticulum and mitochondrial dysfunction in ECs, thus affecting endothelial function. Nevertheless, the exact driving mechanisms require further investigation.

In addition to studies on the intrinsic function of ECs, Wang et al. [104] found three differentially expressed proteins by studying proteins enriched on the secretory EVs of murine adrenal PC12 cells in an oxygen–glucose deprivation (OGD) state. These included scavenger receptor class B member 2 (SCARB2), laminin subunit alpha3 (LAMA3), and LOC100909521 [104]. SCARB2, also known as platelet glycoprotein 4, is a member of the CD36-like superfamily and is an important cell surface and skeletal muscle mitochondrial outer membrane glycoprotein involved in angiogenesis, thrombosis, atherosclerosis, malaria, diabetes, steatosis, dementia, and obesity [105]. LAMA3 is associated with, for example, cell adhesion [106] and EC function and development [107]. With regard to cell adhesion-related functions, Zhan et al. [108] found that ECs could secrete exosomes enriched in heat shock protein 70 (HSP70) and that these exosomes could induce monocyte adhesion to ECs through the action of HSP70. Moreover, MPs secreted by ECs could induce coagulation in vitro via a TF/VII factor-dependent pathway, and are enriched in E-selectin, ICAM-1, αvβ3, and platelet EC adhesion molecule-1 (PECAM-1), with all of these effectors suggested to have adhesion-promoting functions [109].

### Inflammatory Response

Both conventional bacterial-induced and non-bacterial-induced inflammation, and pyroptosis, a specific type of inflammatory death, occupy an important part of the thrombosis process [110]. For example, in bacterial-induced sepsis, the hypercoagulable state of blood is associated with bacterial stimulation of cellular pyroptosis [111]. Here, intracellular inflammatory vesicle activation causes the release of TF (in the form of MVs) to activate the caspase-1-related cellular pyroptosis pathway, thereby inducing systemic coagulation and even death. However, when gasdermin-D (GSDMD) expression is inhibited, the cellular pyroptosis is eliminated and the systemic hypercoagulable state is induced [46]. Wang et al. [112] found that caspase1 substrates and their interacting proteins are mainly localized in various organelles, including the nucleus. This was done using proteomics data analysis, protein interaction, intracellular localization, and gene expression analysis of caspase-1 substrates and several of its interacting proteins. Caspase-1 cleaves its substrates through cytosolic-associated paracrine pathways, including exosomes, and affects adjacent cell focalization. In response to inflammatory stimuli, caspase1 and IL-1β complexes in cells are displaced and encapsulated by cell membrane invaginations and released as EVs, causing subsequent inflammatory responses [113], a release process likely associated with the P2X purinoreceptor 7 (P2X purinoreceptor 7) [114]. As concluded by Boilard et al. [115], PEVs may contain high levels of IL-1β, which in turn promotes an inflammatory response in the joint cavity of patients with arthritis. In addition, PEV can lead to cascade reactions in adjacent platelets and ECs through enriched arachidonic acid, which in turn produces thromboxane A2 and cyclooxygenase 2 (COX2) [116], whereas macrophage-derived and dendritic cell (DC)-derived exosomes can also enrich leukotriene synthase, causing target cells to produce pro-inflammatory leukotriene B4 and leukotriene C4, which in turn induce an inflammatory response [117].

Apoptotic vesicles are extremely widespread in inflammation research. It has been suggested that apoptotic vesicles secreted by ECs contain IL-1α and its precursors and that these apoptotic vesicles induce monocytes to secrete chemokine-1 and IL-8 chemokines in an IL-1α-dependent (but not an IL-1β-independent) manner, thereby mediating the development of aseptic inflammation [118]. Moreover, EVs secreted by ECs can stimulate ECs to specifically bind monocytes through the oxidized phospholipids (POVPC) enriched within them, which in turn induces a subsequent inflammatory response [119]. Baharak et al. [120] found that EVs secreted by ECs under inflammatory stimulation contain various types of pro-inflammatory factors, including ICAM-1, C–C motif chemokine ligand 2 (CCL-2), IL-6, IL-8, CXCL-10, CCL-5, and TNF-α. The EVs selectively transferred these inflammatory mediators to target cells and regulated the anti-inflammatory/pro-inflammatory phenotype switch in ECs and monocytes [120].

**Fig. 3:**
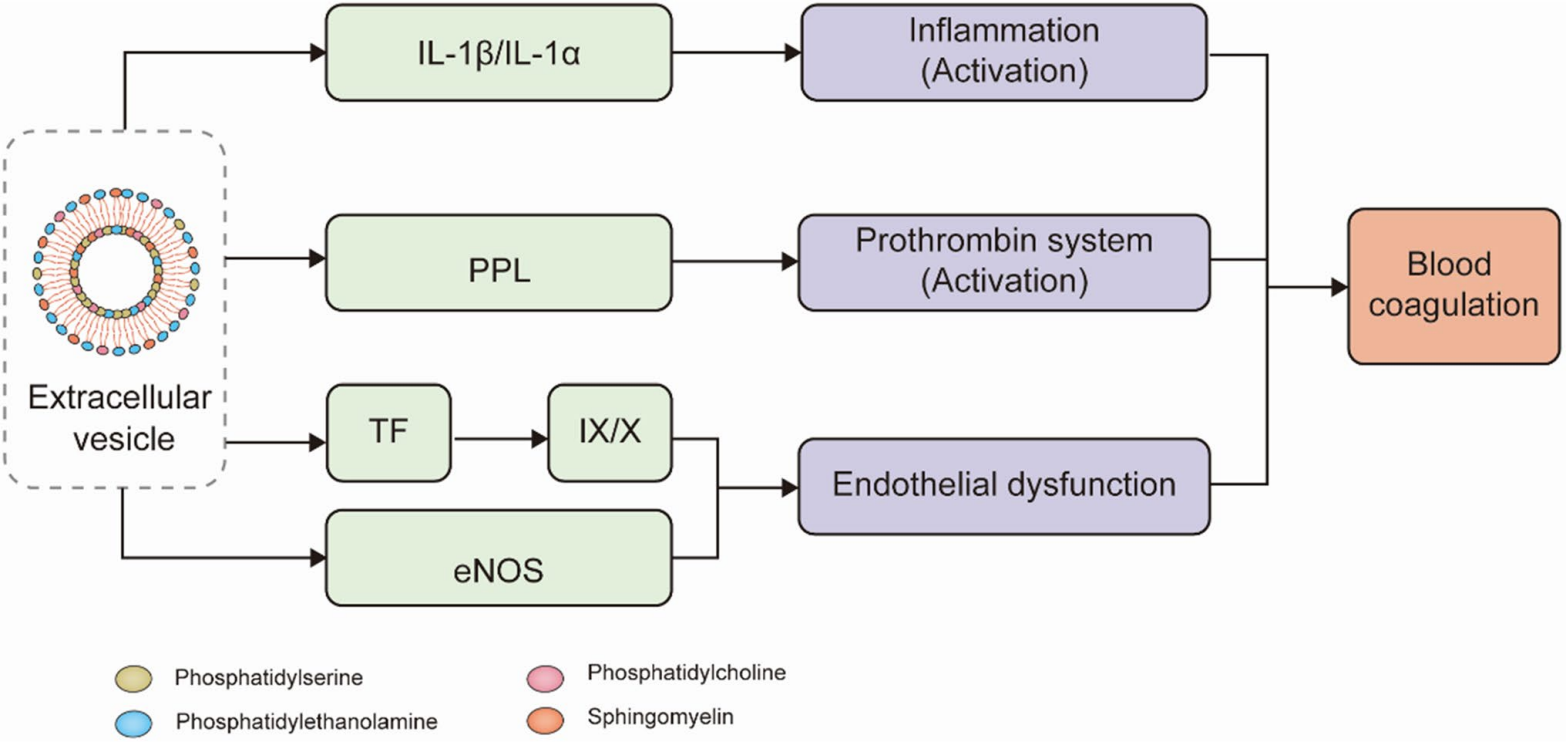
Diagram illustrating the mechanism of blood coagulation caused by extracellular vesicles through various substances

Various types of bacteria associated with inflammation can also secrete EVs, and depending on the bacterial species, they may be enriched with different effector proteins, thereby inducing different inflammatory effects. For example, *Porphyromonas gingivalis* secrete vesicles carrying their specific immunogenic antigens, which in turn induce mucosal immune responses [121]. Moreover, *Helicobacter pylori*, *Pseudomonas aeruginosa*, and *Neisseria gonorrhoeae* secrete EVs enriched with a bacterial-specific peptidoglycan, which activates NF-κB-related inflammatory systems in epithelial cells, thereby inducing an inflammatory response [122]. However, these processes are mostly specific to bacteria and are not the focus of this review. Figure 3 shows an abbreviated schematic to illustrate the approximate process of EV-induced coagulation.

### Non-Coding RNAs

Non-coding RNAs are widely involved in a variety of phenomena, with approximately 10–30% of human genome expression being regulated by miRNAs [123]. Most miRNA-based studies are based on high-throughput microarray analysis of various diseases. Plasma miRNA analysis of patients with coronary artery disease found that miR-19b was more differentially expressed in patients with coronary artery disease compared to that in normal subjects [124]. In a subsequent study, it was confirmed that miR-19b binds to the mRNA of TF, which in turn regulates coagulation function. In vivo, miR-223, miR-339, and miR-21 are enriched in PMPs after TF activation of the prothrombin system, which may then allow miR-223 to regulate the corresponding function of ICAM-1 by inhibiting the phosphorylation of p38, JNK, and ERK, and blocking the nuclear translocation of NF-κB p65 [125]. In addition, miR-223 may also exist as argonaute 2 (Ago2)-miR-223 complexes that are able to regulate the expression of FBXW7 and EFNA1 in ECs, which in turn affect their function. They may heterotypically regulate gene expression in ECs and other receptor cells of the circulatory system, perhaps with a corresponding effect on thrombus formation [126]. In addition, PMP-miR-223 has been shown to regulate arterial thrombosis after endothelial injury by affecting insulin-like growth factor receptor-1 (IGF-1R) in the vascular wall [127]. miR-1915-3p enriched within PMPs can also inhibit the expression levels of Rho GTPase family member B in target cells, thereby inducing megakaryopoiesis and thus promoting platelet-associated thrombopoiesis, a process that does not cause altered levels of thrombopoietin (TPO) [128]. Exosomes secreted by breast, colon, and hepatocellular carcinoma cells are enriched for miR-21 and promote proliferation, migration, and invasion of endothelial cells by targeting IL-6 receptors within endothelial progenitor cells, which in turn mediates venous thrombosis [129]. Conversely, a study of patients with cardiopulmonary disease continuously exposed to particulate air pollution (PM) found that 17 EV-miRNAs had increased expression in serum following PM exposure. Among these, miR-302b, miR-200c, and miR-30d were associated with inflammation and the coagulation system [130].

miRNA matrix analysis of exosomes from an LPS-induced rat DIC model found that of the 46 miRNAs with upregulated expression, seven (miR-21, miR-16, miR-26a, miR-26b, miR-23a, miR-23b, and miR-126) were enriched in human platelets [131, 132]. Moreover, some of the 46 overexpressed miRNAs targeted, or had effects on, cell adhesion and coagulation-related proteins (e.g., sICAM-1, E selectin, and fibrinogen α chain; Table 2), suggesting that miRNAs in MVs may play a role in the coagulation system [133]. MicroRNA microarray analysis of patients with lower extremity venous thrombosis detected 97 miRNAs which compared to the healthy group. Among them, miR-10b-5p, miR-320a, miR-320b, miR-424-5p, and miR-423-5p expression was upregulated, and miR-103a-3p, miR-191-5p, miR-301a-3p, and 199b-3p expression downregulated in the EVs of plasma from the patients (Table 3).

**Table 2:** Coagulation-related protein targets corresponding to serum miRNAs in rats according to the DIC model [146]

Predicted target genes	miRNA name gene
sICAM-1	miR-23b, miR-27a,miR-99a,miR-100,miR-324-5p, miR-363
PAI-1	miR-30a, miR-30d,miR-182,miR-384-5p
E Selectin	miR-16, miR-195
Alpha chain of fibrinogen	miR-29c

**Table 3:** Protein targets corresponding to each miRNA in plasma EVs of VTE patients [133]

miRNA name gene	Predicted target genes
hsa-miR-10b-5p	CADM2, PPARA, CALCR, CDK6, MAP3K7, MAP3K2, PIK3CA, CAMK2B
hsa-miR-320b	ICAM1, PDK3, PIK3R1, ITGB3
hsa-miR-320a	CD61, THBS1, HIF1a, MAPK1, IGF1, IGFR
hsa-424-5p	CD61, THBS1, HIF1a, MAPK1, IGF1, IGFR
hsa-miR-423-5p	VEGFB, NOS3, ADMTS4, CDH1, THBS3, HIF3A, CADM3, CAM1, AK1
hsa-miR-103a-3p	THSD7A, PAFAH1B2, ADAMTSL3, CD49B PRKCE, PRKG1, CAMK2G, CAMKK1, PRKAB, ADAMTSL2, DICER1, PDE8B, PLCA2G2F, PDE3B, PLCB1, PIKs3R1, FIGF
hsa-miR-191-5p	PLCD1, NFIA
hsa-miR-301a3p	TSC1, TBXAS1, ADAMTS18, ADAMTS19, EDN1, LDLR, PPARG, BMPR2, ADCY1, ADCY2, PDGFR, TGFBR, PDK1
has-miR-199b-3p	TSC1, TBXAS1, ADAMTS18, ADAMTS19, EDN1, LDLR, PPARG, BMPR2, ADCY1, ADCY2, PDGFR, TGFBR, PDK1

Long non-coding RNAs (lncRNAs) and circulating RNAs (circRNAs) have also received attention. In an exosome screen of circulating sera from 79 patients with hepatocellular carcinoma, miRNA-21 and lncRNA-ATB were found to be directly related to possible portal vein thrombosis [134]. Moreover, a study of serum exosomes from patients with acute coronary syndrome revealed that stimulation of ECs by these exosomes prompted the enrichment of circRNA0006896 by ECs, with this circRNA regulating EC behavior by targeting and inhibiting miR1264 and SOCS3 expression, and upregulating DNMT1 and phosphorylated STAT3 levels, which in turn play a role in carotid artery plaque development [135].

#### Other Effect Components

As mentioned earlier, various types of extracellular vesicles have a complex composition in vivo and may contain other effectors in addition to proteins and coding and non-coding RNAs. For example, tumor cell-derived EVs carry a large amount of genomic DNA (gDNA), or even mutated gDNA (HRAS and HER2), which may lead to cancer-related thrombogenic effects [136]. Likewise, EVs secreted by human adipose mesenchymal stem cells carry substances that promote the enrichment of large amounts of elastin and collagen within vascular scaffolds, the exact mechanism of which is not yet clear [137].

In addition, extracellular ATP signals can cause cellular inflammatory and coagulation effects in addition to stimulating the production of EVs by cells [45]. Because EVs may contain ATP signals within themselves [138], these ATP signals not only prevent the internalization of TF and PS but also induce the conversion of TF to a conformation with a high affinity for its ligand coagulation factor VII, which leads to procoagulant effects [139]. Moreover, nicotinamide adenine dinucleotide (NADH) and NADPH have been shown to be potentially present in EVs [140] and may be involved in thrombosis through the regulation of NOX-1 [94] and NOX-2 complexes [38].

## Summary and Prospects

The aim of this review was to summarize and describe the effect of EVs secreted by various cells on thrombosis to facilitate the future development of a precise therapy for thrombosis involving vesicles. Notably, some studies have already been initiated targeting this effect in the field of tissue engineering. For example, Pawlowski et al. [141] developed PMPs-inspired nanovesicles (PMIN) using a liposomal platform that protects circulating encapsulated thrombolytic drugs from targeted uptake and action, actively anchoring them to thrombi through molecular mechanisms associated with PMPs, and releasing them through thrombus-associated enzymatic triggers. Their study also demonstrated that intravenous delivery of thrombolytic-loaded PMIN could achieve targeted fibrinolysis without compromising systemic hemostasis in vivo. In addition to thrombolytic therapy, Hou et al. [142] showed that exosomal surface-coated stent technology significantly increased the number of ECs in the stent lining while also improving endothelial function at the stent contact surface, contributed to endothelialization of the stent, and reduced thrombosis relative to conventional arterial stents. In addition, researchers have also begun to study drugs targeting PEVs. Cilostazol, a new antithrombotic drug, was found to specifically reduce the release of PS-positive EVs from human platelets while not affecting total EV release [143]. We believe that this is a promising research direction.

In addition, the expression of miR-223, miR-339, and miR-21 enriched within EVs was significantly increased in an in vivo thrombosis model [144]. Moreover, circRNA-0006896 is enriched within serum-derived EVs from patients with myocardial infarction and stimulates vascular endothelial cell proliferation and migration [135]. Combined with the relationship between miR-23b, miR-10b-5p, and other miRNAs and thrombosis-related proteins, as illustrated in Tables 2 and 3, these results suggest the novel possibility that non-coding RNAs in EVs may serve as biomarkers to predict acute thrombus formation after atherosclerosis. The combination of such new research techniques and methods along with the performance of more in-depth studies will establish a new foundation for cardiovascular research. Especially in recent years, with the global outbreak of COVID-19, thromboembolism, as a very serious complication of COVID-19, has also been gradually taken into account by researchers. As mentioned in this paper, TF-positive EVs are abundantly expressed in the circulation of COVID-19-infected patients [145]. We anticipate that this review will establish a new horizon in the field of cardiovascular research and acute thrombosis treatment, especially with regard to thrombosis caused by EVs secreted by individual cells. Moreover, as more emerging technologies are being developed, new diagnostic and therapeutic strategies related to EVs are expected to also be identified for related diseases in the future.
